# Integrated Genomic and GEO Data Analysis Reveals Therapeutic Targets for Rosacea

**DOI:** 10.1111/jocd.70334

**Published:** 2025-07-10

**Authors:** Xinrui Deng, Sui He, Huiyu Long, Jinyuan Xiao, Zhengchun Xie

**Affiliations:** ^1^ Hunan University Affiliated Xiangtan Central Hospital, Clinical Laboratory Xiangtan Hunan China; ^2^ Xiangya School of Pharmaceutical Sciences, Central South University Changsha Hunan China

**Keywords:** druggable genes, GWAS, IRF1, mendelian randomization, rosacea, SLC22A5

## Abstract

**Background:**

Rosacea is a chronic inflammatory facial disorder with limited therapeutic options, severely impacting patients' quality of life. The identification of druggable genes plays a crucial role in facilitating the development of effective therapeutic strategies.

**Methods:**

We conducted Mendelian randomization (MR) and Summary‐based Mendelian randomization (SMR) analyses by integrating data on 5883 druggable genes, cis‐expressed quantitative trait loci (eQTL) from blood and skin tissue (lower leg and suprapubic), and genome‐wide association study (GWAS) data on rosacea to elucidate the causal relationship between druggable genes and rosacea. Robustness was confirmed via heterogeneity/horizontal pleiotropy tests, Steiger filtering, Bayesian colocalization analysis, and the heterogeneity in dependent instruments (HEIDI) analysis. The expression levels of identified druggable genes were validated using the GSE65914 data sets. Further analyses included protein–protein interactions (PPIs), functional enrichment analysis, phenome‐wide association study (PheWAS), drug prediction, and molecular docking.

**Results:**

MR and SMR analyses identified IRF1 and SLC22A5 as druggable genes for rosacea, with Bayesian colocalization strongly supporting shared causal variants. GEO data sets confirmed significant upregulation of IRF1 and downregulation of SLC22A5 in rosacea patients. PPIs and functional enrichment analyses revealed that IRF1 promotes inflammation by regulating immune cell activation and interferon signaling pathways; SLC22A5 regulates membrane transport and metabolic processes, and its dysregulation may lead to lipid homeostasis imbalance. PheWAS analysis indicated no other phenotypes associated with IRF1 and SLC22A5. Drug prediction and molecular docking verified the pharmacological value of IRF1 and SLC22A5.

**Conclusion:**

This study identified IRF1 and SLC22A5 as potential drug targets for the treatment of rosacea, and their significant therapeutic potential provides a critical foundation for the development of targeted therapies.

## 1 Introduction

Rosacea is a chronic inflammatory disorder characterized by facial erythema, blood vessel dilation, pimples, and pustules, potentially accompanied by ocular symptoms, with four clinical subtypes: erythematotelangiectasia rosacea (ETR), papulopustular rosacea (PPR), phymatous rosacea (PhR), and ocular rosacea (OR) [1, 2]. The exact cause is unknown, but it may be connected with various conditions, such as cardiovascular, gastrointestinal, neurological, psychiatric, and autoimmune diseases, as well as certain malignant tumors [3]. There is currently no cure for rosacea, and its impact on facial appearance can lead to severe psychological distress, including anxiety and depression. This not only diminishes patients' quality of life but also results in social withdrawal [4]. Although topical treatments and oral tetracyclines are currently available, treatment failures and side‐effect concerns emphasize the critical demand for new therapeutic targets [5].

In recent years, genome‐wide association studies (GWAS) have effectively identified several common genetic variants related to the progression of rosacea [6, 7], These findings offer novel insights into the disease's pathological mechanisms; however, translating these genetic discoveries into actionable therapeutic targets remains a significant challenge [8]. Mendelian randomization (MR), a method that can reveal causality between exposure and outcome by using genetic variants as instrumental variables (IVs), has been extensively employed in drug target discovery and the repurposing of drugs [9]. Expression trait loci (eQTLs) are used as IVs in the MR analysis of drug targets and are analyzed with disease GWAS data sets to determine whether the targets have an effect on the disease at the genetic level [10]. Currently, no study has comprehensively analyzed the potentially druggable genes with rosacea.

In this research, we integrated eQTL data from three different tissues and GWAS data of rosacea in European populations to systematically evaluate druggable genes associated with rosacea. The analytical framework included two‐sample MR analysis, SMR, and HEIDI test, as well as Bayesian colocalization analysis. These approaches not only evaluated the causal relationship between the druggable genes and rosacea but also ensured the robustness of the results through various sensitivity analyses. Furthermore, the expression levels of key druggable genes were verified by GSE65914 data sets; PPI network analysis and functional enrichment analysis were used to explore the molecular interaction network and functional synergy mechanism of key druggable genes related to disease. Finally, PheWAS association analysis, drug prediction, and molecular docking were utilized to validate the therapeutic potential of identified druggable genes. Our results provide a new theoretical basis for elucidating the pathogenesis of rosacea and lay an important foundation for the subsequent development of targeted drugs and clinical translation.

## 2 Methods

### 2.1 Druggable Genes

**FIGURE 1:**
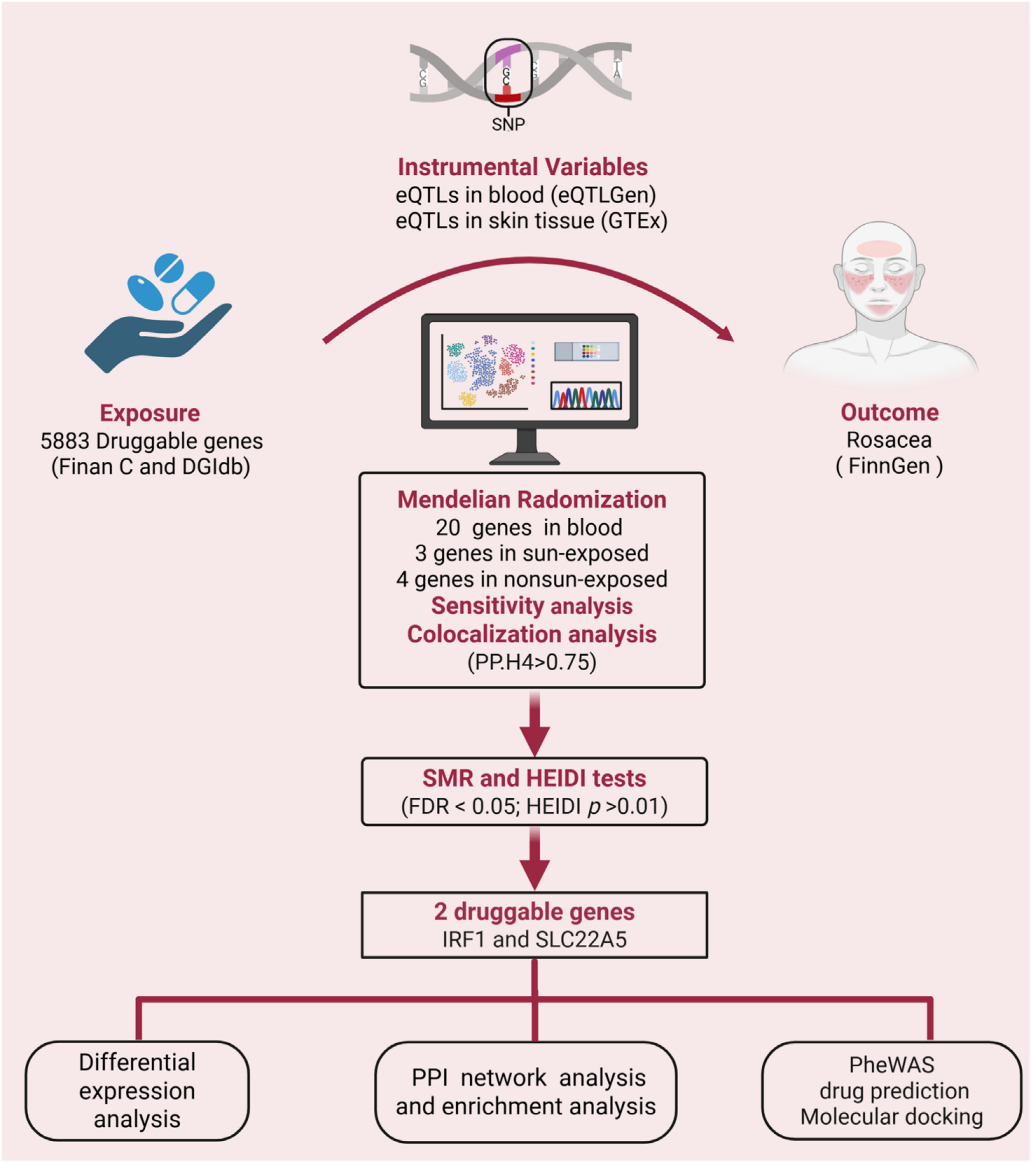
Overview of study design to identify drug targets for rosacea (created with Biorender.com).

Figure 1 illustrates the research workflow. To ensure that the druggable genes have scientific reliability and promising therapeutic potential, the druggable genes were derived from the Drug‐gene Interaction Database (DGIdb v5.0, https://www.dgidb.org/downloads) [11], and the review by Finan C et al. [12] By integrating the druggable genes from these two sources, a total of 5883 unique genes, approved by the Human Genome Organization's Committee on Gene Nomenclature, were compiled for subsequent analysis (Table S2).

### 2.2 Data Source

The blood eQTL data set was retrieved from eQTLGen (https://eqtlgen.org/) [13], which includes cis‐eQTL information for 19 250 genes from 31 684 individuals. Most of the participants were of European descent. Additionally, eQTL data of skin tissue were obtained from the Genotype‐Tissue Expression (GTEx v8.0, http://www.gtexportal.org) [14] consortium. The GWAS data of rosacea were sourced from FinnGen Release 11 (https://www.finngen.fi/en) [15], which includes a group of 2808 cases and 432 686 controls.

### 2.3 Mendelian Randomization and Sensitivity Analysis

Two‐sample MR analysis was performed using the “TwoSampleMR” R package (version 0.6.0) [16]. To construct the IVs, the screening process was conducted according to the following criteria: (1) The SNP demonstrated a reliable correlation with the level of gene expression (*p* < 5E‐08), (2) IVs were clumped within a genetic window of 500 kb using a strict linkage disequilibrium (LD) threshold of *r^2^* = 0.1, indicating partial independence between each selected SNP, and (3) SNPs with weak instrument strength (*F*‐statistic < 10) were excluded to prevent potentialbias from instrumental variables with inadequate statistical power.

When only one SNP was available for analysis, we used the Wald ratio method for MR estimation; in cases with multiple SNPs, we applied the IVW method with random effects. The P‐values of druggable genes were adjusted using the Bonferroni correction, with thresholds for eQTL in blood (0.05/3211), eQTL in sun‐exposed skin of the lower leg (0.05/1231) and in sun‐not‐exposed suprapubic skin (0.05/1063), respectively. To validate primary MR results, we performed sensitivity analyses using the weighted median method and MR‐Egger. Cochran's Q test and MR Egger intercept test were used to evaluate potential heterogeneity and horizontal pleiotropy (*p* > 0.05), for genes with more than two instruments. Last, Steiger filtering was used to test for reverse causality, a ‘TRUE’ direction with *p* < 0.05 indicates no significant reverse causality.

### 2.4 SMR and HEIDI Test

We performed the analysis using SMR software (version 1.3.1), and results with false discovery rate (FDR) correction of less than 0.05 were considered significant. Moreover, we applied the HEIDI (tool‐dependent heterogeneity) test to evaluate whether the observed associations might be influenced by linkage disequilibrium. Associations with a HEIDI test *p*‐value below 0.01 indicated that the observed association could be due to linkage disequilibrium [17, 18]. The integration of SMR and HEIDI tests added further evidence supporting the association between druggable gene eQTL data and rosacea.

### 2.5 Bayesian Colocalization Analysis

The “coloc” R software package (version 5.2.3) was applied for the colocalization analysis of druggable genes that had shown significant associations through MR analysis [19]. This analysis employed a Bayesian approach to investigate whether the relationship between druggable genes expression and rosacea was driven by common causal genetic variants. Using the default prior probabilities, we set P1 = P2 = 1 × 10^−4^ to indicate the probability that an SNP was associated with either trait 1 or trait 2. Additionally, we set P12 = 1 × 10^−5^ to represent the probability that an SNP contributes to the causal effect of both traits. The significance threshold for colocalization was established as PPH4 greater than 0.75, indicating that genes that demonstrate colocalization with rosacea at this level of confidence could be considered potential drug target genes.

### 2.6 Gene Expression Omnibus (GEO) Analysis

The microarray data sets GSE65914, which contain gene expression profiles of 19 patients with rosacea and 10 healthy controls, were obtained from the GEO database. After normalization, differentially expressed genes (DEGs) were identified using the GEO2R online tool (https://www.ncbi.nlm.nih.gov/geo/geo2r/), with the cutoff criteria set at an adjusted *p* < 0.05 and |logFC| ≥ 1.

### 2.7 PPI Analysis and Enrichment Analysis

The GeneMANIA platform (https://genemania.org/) [20] was utilized to construct a protein interaction network of key druggable genes of rosacea. The DAVID database was used to annotate the functions of the key targets, and gene ontology (GO) functional analysis and Kyoto Encyclopedia of Genes and Genomes (KEGG) pathway enrichment analysis were performed based on the R platform (*p* < 0.05) [21].

### 2.8 Phenome‐Wide Association Analysis

The AstraZeneca PheWAS Portal (https://azphewas.com/) was a public repository for gene–phenotype associations [22], capable of performing comprehensive PheWAS studies to further assess the levels of pleiotropy and potential side effects of prospective drug targets.

### 2.9 Candidate Drug Prediction

The Drug Signatures Database (DSigDB, http://disgdb.tanlab.org/DSigDBv1.0/) consists of a compilation of drug and small molecule‐associated gene sets, which are based on data regarding quantitative inhibition and/or drug‐induced gene expression alterations [23]. We analyzed the identified significant druggable genes using the DSigDB drug database on the Enrichment platform (https://maayanlab.cloud/modEnrichr/) [24] to pinpoint potential targeted drugs, predict candidate medications, and assess the pharmacological activity of these genes.

### 2.10 Molecular Docking

Molecular docking was used to evaluate the binding properties of core active ingredients to key targets [25]. The two‐dimensional structures of each small molecule ligand are obtained from the PubChem database (https://pubchem.ncbi.nlm.nih.gov/). For the structure of the target genes encoding proteins, data structure is downloaded from the AlphaFold database (https://alphafold.ebi.ac.uk/) Using Autodock v4.2.6 [26], we docked the top five drug candidates with their targets, with results visualized in PyMOL (https://www.pymol.org/).

## 3 Results

### 3.1 Druggable Genes Associated With Rosacea MR Analysis

**FIGURE 2:**
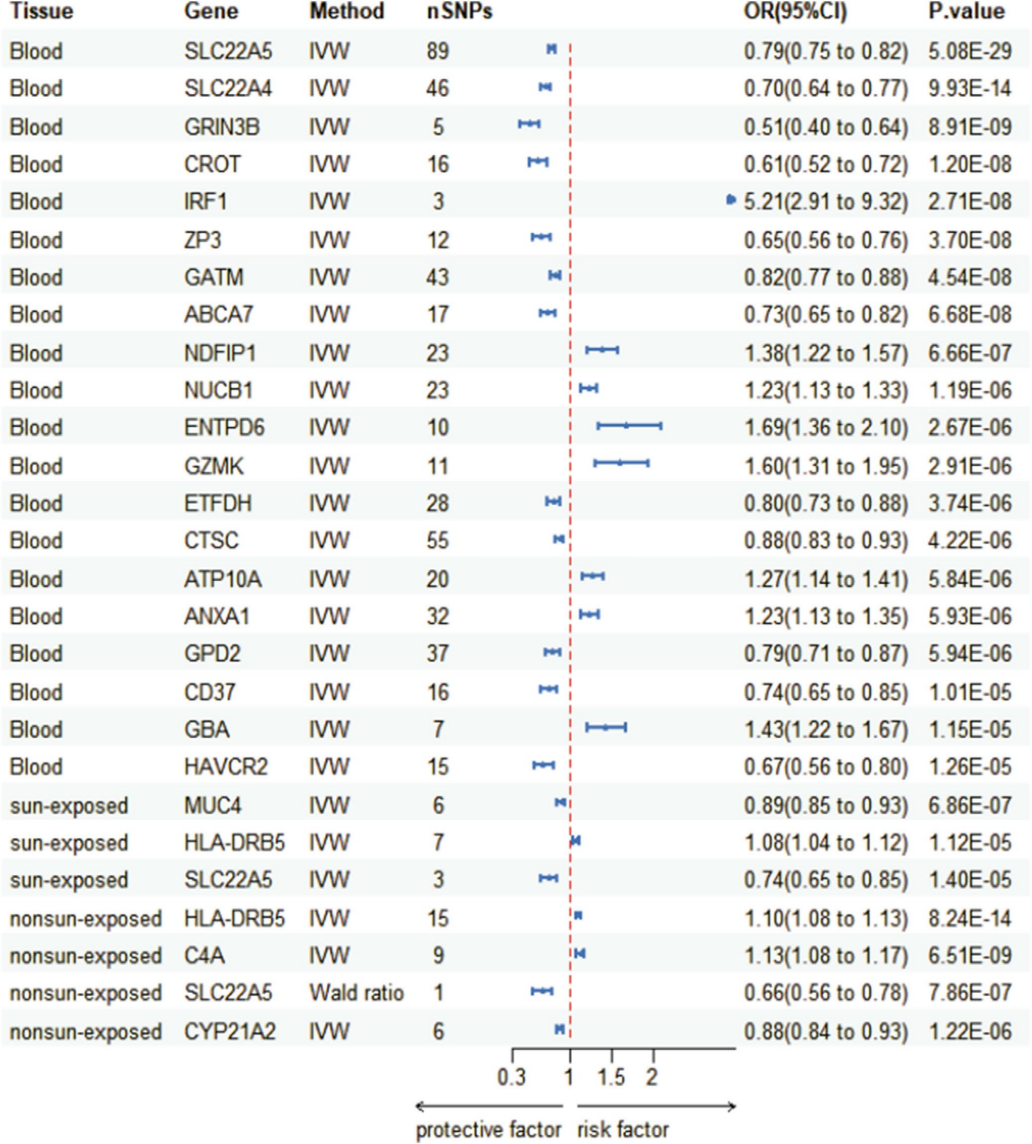
Significant MR analysis results. CI, confidence interval; IVW, inverse variance weighted; nonsun‐exposed, sun‐not‐exposed suprapubic skin; SNP, single nucleotide polymorphism; sun‐exposed, sun‐exposed skin of the lower leg.

In the IVW and Wald ration method, we identified a total of 20 potential druggable genes in the blood (*p* < 1.54E‐05), 3 in sun‐exposed skin of the lower leg (*p* < 4.06E‐05), and 4 in nonsun‐exposed suprapubic skin (*p* < 4.70E‐05) that were significantly associated with rosacea (Figures 2 and S1). The analysis revealed that potential drug targets passed the tests (*p* > 0.05), except for SLC22A4 and GATM, which exhibited signs of heterogeneity or horizontal pleiotropy. The Steiger filtering test supported our hypothesis that IVs caused the outcome variable and not the other way around (*p* < 0.05). The *p*‐value for HLA‐DRB5 was recorded as “NA” and thus was not considered in subsequent analyses. Detailed results for the significant IVs and the comprehensive results of the MR analysis are presented in Tables S3–S8.

### 3.2 SMR Analysis and HEIDI Test

Through the comprehensive analysis of rosacea of three tissues, SMR analysis identified three druggable genes in human blood that were causally associated with rosacea‐related traits (*p*‐FDR < 0.05), including P4HA2, IRF1, and SLC22A5. HEIDI tests showed that the association between expression levels of IRF1, SLC22A5, and rosacea was not caused by linkage imbalance (*p* > 0.01) [18]. Additionally, in the eQTL data sets from sun‐exposed skin tissues, two druggable genes that were causally linked to rosacea‐related traits failed to pass the HEIDI test (Figure S2 and Table S9).

### 3.3 Colocalization Analysis

To determine whether the genetic variants that affect the exposure variable also have a causal impact on the outcome variables through a shared biological pathway, we performed colocalization analyses of results derived from the MR and SMR. Results indicated that IRF1 passed the colocalization analysis (PP.H4 = 0.973). SLC22A5 revealed varying degrees of evidence of colocalization in the three tissues: for the blood, PP.H4 was 0.860; for the sun‐exposed tissue, PP.H4 was 0.809; for the nonsun‐exposed tissue, PP.H4 was 0.781 (PP.H4 > 0.75 was strong evidence of colocalization) (Table 1).

**TABLE 1:** Colocalization results of IRF1 and SLC22A5.

Gene	Tissue	PP.H0	PP.H1	PP.H2	PP.H3	PP.H4
IRF1	Blood	2.6E‐21	0.000011	6.51E‐18	0.027	0.973
SLC22A5	Blood	1.02E‐199	0.000056	2.55E‐196	0.140	0.860
SLC22A5	Sun‐exposed	1.25E‐47	0.000075	3.21E‐44	0.191	0.809
SLC22A5	Nonsun‐exposed	1.07E‐30	0.000085	2.75E‐27	0.219	0.781

*Note:* PP.H0‐PP.H4 denote the posterior probabilities of various hypotheses, with a PP.H4 > 0.75 considered to indicate a significant colocalization.

Abbreviations: nonsun‐exposed, sun not exposed suprapubic skin; sun‐exposed, sun‐exposed skin of the lower leg.

### 3.4 Differential Expression Analysis

**FIGURE 3:**
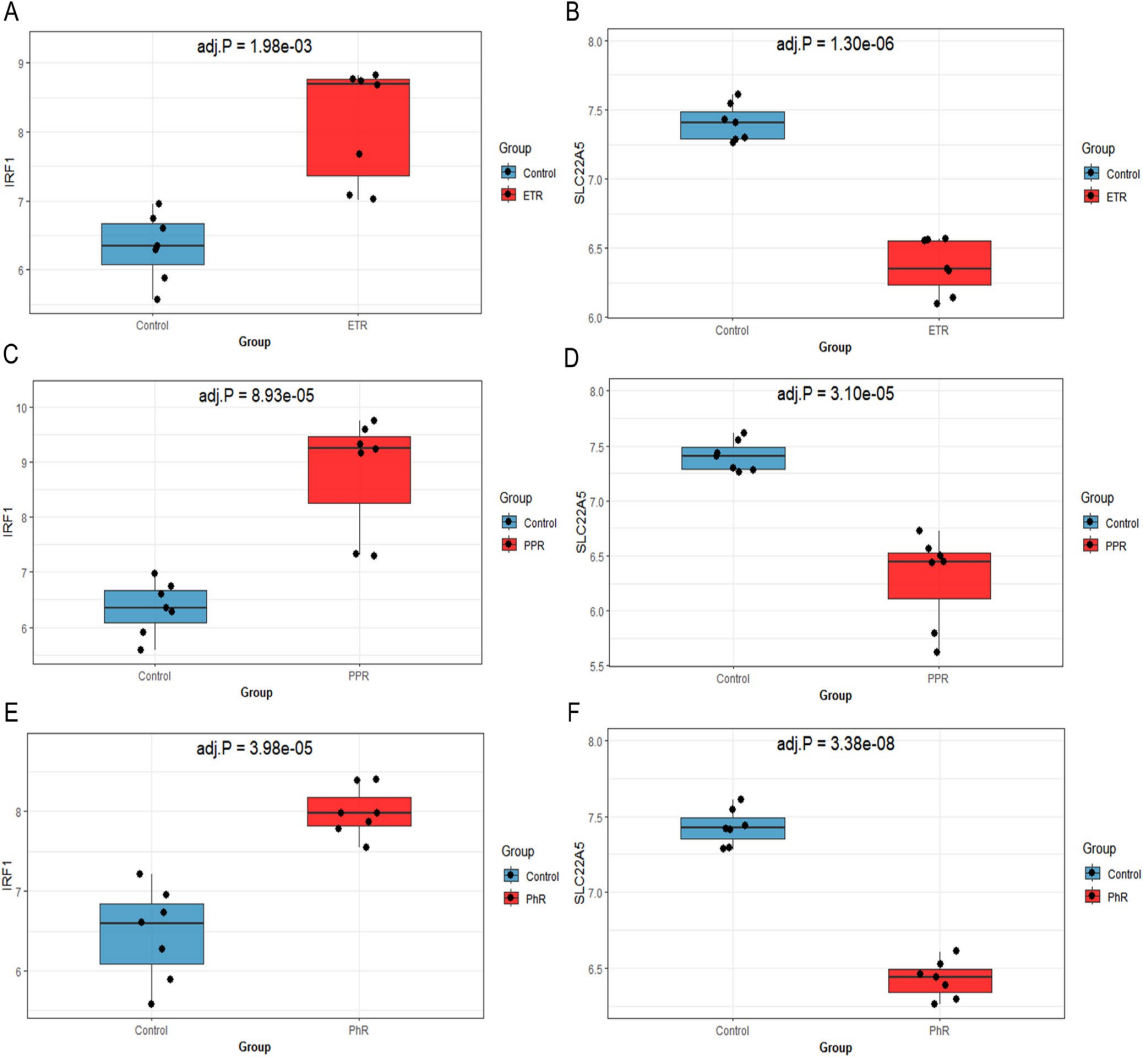
Expression levels of IRF1 and SLC22A5 in rosacea. (A) IRF in ETR (B) SLC22A5 in ETR (C) IRF in PPR (D) SLC22A5 in PPR (E) IRF in PhR (F) SLC22A5 in PhR ETR, Erythematotelangiectasia rosacea; PhR, Phymatous rosacea; PPR, Papulopustular rosacea.

By analyzing the GSE65914 data sets, we evaluated the expression of IRF1 and SLC22A5 genes in the three major subtypes of rosacea. IRF1 was significantly upregulated in ETR, PPR, and PhR. In contrast, SLC22A5 expression was significantly downregulated in all three subtypes (*adj.p* < 0.05, |logFC| ≥ 1). This result validated the expression of IRF1 and SLC22A5 in patients with rosacea and further supported their potential therapeutic value (Figure 3 and Table S10).

### 3.5 PPI Analysis and Enrichment Analysis

**FIGURE 4:**
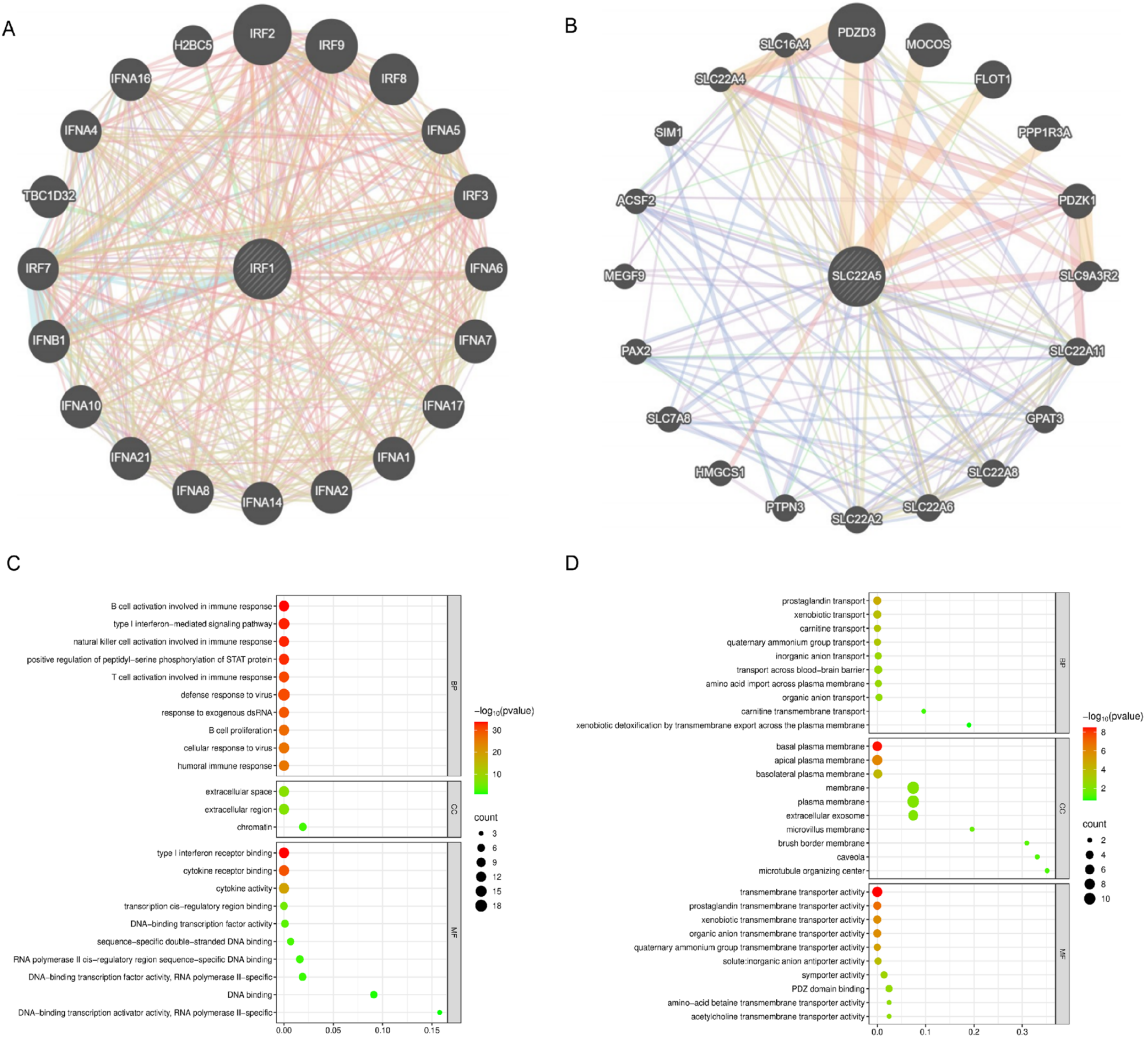
PPI analysis and GO function enrichment analysis. (A) GeneMANIA gene network of IRF1 (B) GeneMANIA gene network of SLC22A5 (C) GO functional enrichment analysis for IRF1‐centered network (D) GO functional enrichment analysis for SLC22A5‐centered network.

Based on the GeneMANIA database, gene interaction networks were constructed with IRF1 and SLC22A5 as central linker genes, respectively. In the network centered on IRF1, GO enrichment analysis demonstrated that IRF1 was primarily associated with immune cell activation, regulation of immune responses, and type I interferon signaling pathways. It plays a critical role in RIG‐I‐like receptor signaling, Toll‐like receptor signaling, and cytosolic DNA‐sensing pathways. The network centered on SLC22A5 revealed its significant involvement in membrane transport functions and various metabolic processes, including signaling pathways related to choline metabolism, folate transport and metabolism (Figure 4 and Table S11 and Table S12).

### 3.6 Phenome‐Wide Association Analysis

We performed a phenome‐wide MR study via the AstraZeneca PheWAS portal to investigate the potential side effects of IRF1 and SLC22A5. The results showed that there was no evidence of a significant relationship between these two drug targets and other phenotypes in the PheWAS Portal at a genome‐wide significance level (*p* < 5E‐08). This strengthens the validity of the findings, suggesting that drugs targeting IRF1 and SLC22A5 have no potential adverse effects. Figure S3 shows the complete result.

### 3.7 Candidate Drug Prediction

Evaluating the interactions between proteins and drugs is crucial in determining whether target genes can be viable candidates for drug targeting. The results revealed that the top four drugs significantly associated with both IRF1 and SLC22A5 were Tretinoin (MCF7 UP), Cephaeline (PC3 UP), Amiodarone (CTD 00005381), and Emetine (PC3 UP). Additionally, Puromycin (CTD 00006652), Tosyllysyl chloromethane (CTD 00006909), and Ro 41‐5253 (CTD 00003040) were associated with IRF1, as was Ticlopidine (MCF7 UP). Furthermore, TTNPB (MCF7 UP) and Tetraethylammonium hydroxide (BOSS) were discovered to be related to SLC22A5 (Table 2).

**TABLE 2:** Candidate drugs for IRF1 and SLC22A5 were predicted using DSigDB.

Term	*p*	Adjusted *p*	Genes
Tretinoin	2.28E‐05	0.003	IRF1; SLC22A5
Cephaeline	6.59E‐04	0.024	IRF1; SLC22A5
Amiodarone	9.75E‐04	0.024	IRF1; SLC22A5
Emetine	0.0016	0.024	IRF1; SLC22A5
Puromycin	0.0016	0.024	IRF1
Tosyllysyl chloromethane	0.0016	0.024	IRF1
Ro 41‐5253	0.0017	0.024	IRF1
TTNPB	0.0019	0.024	SLC22A5
Ticlopidine	0.0021	0.024	IRF1
Tetraethylammonium hydroxide	0.0022	0.024	SLC22A5

### 3.8 Molecular Docking

**FIGURE 5:**
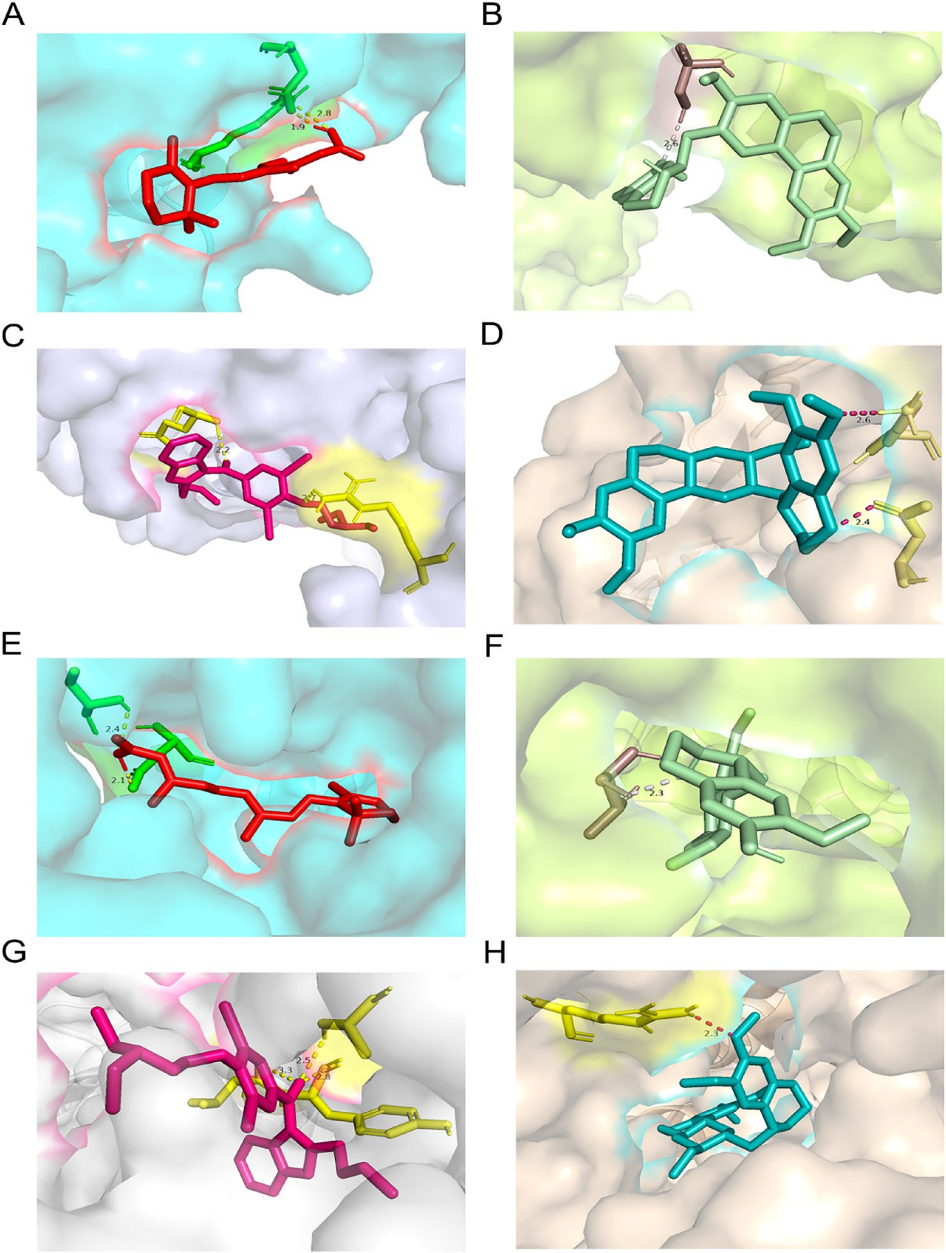
Docking results of available proteins small molecules. (A) IRF1 with Tretinoin, (B) IRF1 with Cephaeline, (C) IRF1 with Amiodarone, (D) IRF1 with Emetine, (E) SLC22A5 with Tretinoin, (F) SLC22A5 with Cephaeline, (G) SLC22A5 with Amiodarone, (H) SLC22A5 with Emetine.

AutoDock 4.2.6 was utilized to assess the affinity of drug candidates, thereby evaluating the potential pharmacological properties of the target and investigating the interactions between the top four drug candidates and the proteins produced by the corresponding genes. The results indicated effective docking of proteins with drugs, as shown in Figure 5 and Table 3. Of note, the interaction between SLC22A5 and Tretinoin is characterized by the lowest binding energy, −6.51 kcal/mol, suggesting highly stable binding and a potential candidate for drug development.

**TABLE 3:** Molecular docking results of IRF1 and SLC22A5 with potential drug candidates.

Target	Structure ID	Drug	PubChem ID	Binding energy (kcal/mol)
IRF1	AF‐P10914‐F1‐v4	Tretinoin	444 795	−4.66
IRF1	AF‐P10914‐F1‐v4	Cephaeline	442 195	−4.33
IRF1	AF‐P10914‐F1‐v4	Amiodarone	2157	−3.30
IRF1	AF‐P10914‐F1‐v4	Emetine	10 219	−4.37
SLC22A5	AF‐O76082‐F1‐v4	Tretinoin	444 795	−6.51
SLC22A5	AF‐O76082‐F1‐v4	Cephaeline	442 195	−5.91
SLC22A5	AF‐O76082‐F1‐v4	Amiodarone	2157	−5.76
SLC22A5	AF‐O76082‐F1‐v4	Emetine	10 219	−6.03

## 4 Discussion

Rosacea is a chronic inflammatory disorder with complex interactions between genetic makeup and environmental triggers [27, 28]. While GWAS has identified a few susceptibility loci, translating these findings into therapeutic targets remains challenging due to the lack of causal evidence [29, 30]. In this research, we identified an important causal relationship between two druggable genes, IRF1 and SLC22A5, and the development of rosacea. The GEO database results were highly consistent with our genetic findings, and comprehensive results were obtained by subsequent functional gene set analysis. In addition, we investigated the druggability of these genes.

Interferon Regulatory Factor 1 (IRF1) is a member of the Interferon Regulatory Factor family and plays an important role in immune and inflammatory response [31]. Our findings showed a significant positive association with increased expression of IRF1 in the blood (OR = 5.21, *p* = 2.71E‐08) and an elevated risk of rosacea; the strong evidence of colocalization suggested a potential role in the pathogenesis of the disease (PP.H4 = 0.973). Upregulated expression of IRF1 in patients with rosacea was confirmed by GEO data sets. Functional enrichment analyses revealed that IRF1 promotes inflammation by regulating immune cell activation and interferon signaling pathways. IRF1 regulated the IFNγ/STAT1 signaling pathway in keratinocytes and enhanced their interaction with macrophages, which drove the persistent inflammatory response of rosacea and then induced clinical manifestations such as pustules, papules, and heat pain [32]. UV exposure had been reported to activate the epidermal IFNγ/pSTAT1/IRF1 axis in nonmelanoma skin cancer, leading to the upregulation of chemokines (CXCL9, CXCL10, and CCL2) [33]. Although direct evidence of the IRF1 pathway in rosacea requires further validation, this finding suggests that environmental factors (UV exposure) may exacerbate disease progression through IRF1‐dependent mechanisms. To date, no effective strategy currently exists to directly inhibit IRF1 activity. However, inhibitors that modulate the upstream JAK/STAT signaling pathway, such as tofacitinib and ruxolitinib, have demonstrated potential for repurposing due to their indirect suppression of IRF1 expression, especially considering their efficacy against IFN‐driven skin disease [34, 35].

Studies demonstrated that reactive oxygen species (ROS) in keratinocytes contribute to the development of rosacea by mediating vascular changes and inflammatory cascades [36, 37]. Although carnitine derivatives can improve inflammatory skin diseases by repairing mitochondrial function and reducing ROS production, their effects on rosacea still need to be verified [38]. Our research provides new genetic evidence for the protective effect of SLC22A5 in rosacea; the MR results of blood showed that the expression of SLC22A5 was negatively correlated with the risk of rosacea (OR = 0.79, *p* = 5.08E‐29). Skin eQTL analysis confirmed its therapeutic potential (sun‐exposed: OR = 0.74, *p* = 1.40E‐05; nonsun‐exposed: OR = 0.66, *p* = 7.86E‐07), strongly supported by colocalization (PP.H4 > 0.75). The GEO data sets verified that the expression of SLC22A5 was downregulated in patients with rosacea (adj. *p* < 0.05, |logFC| ≥ 1). As a key carnitine transporter, SLC22A5 maintains cellular uptake capacity for carnitine, and carnitine homeostasis is essential for mitochondrial fatty acid oxidation and ROS regulation [39]. Molecular docking revealed that Tretinoin, which is known to improve rosacea by anti‐inflammatory mechanisms, binds SLC22A5 with high affinity (−6.51 kcal/mol). Based on the above evidence, we hypothesize that Tretinoin may improve mitochondrial dysfunction by enhancing SLC22A5‐mediated carnitine uptake; this novel mechanism requires experimental validation.

This study offers valuable insights into the treatment of rosacea by integrating druggable genes and eQTL for the quantitative screening of potential drug targets. Colocalization analysis, differential expression analysis, and functional enrichment analysis were employed to elucidate the underlying biological mechanisms. The PPI network was constructed, and the characteristics of the key druggable genes network were analyzed. PheWAS was used to validate the safety of the identified druggable genes, and potential therapeutic compounds were evaluated through drug prediction and molecular docking.

However, our research has some limitations. First, all the data sets used are derived from European ethnic groups, which means the conclusions may not be broadly applicable to people of other racial or ethnic backgrounds. Second, due to the lack of genetic studies at the protein level, we were unable to confirm whether the protein quantitative trait loci for IRF1 and SLC22A5 were associated with the risk of rosacea. Third, the limitations of public eQTL data, including measurement error, sample size limitation, and tissue specificity, may affect our understanding of the multitissue pathological mechanisms of rosacea. Finally, although our MR Analysis identified IRF1 and SLC22A5 as potential drug targets for rosacea, these findings remain hypothetical at this time and require further validation in experimental models.

## 5 Conclusion

In summary, we confirmed the causal relationship between IRF1 and SLC22A5 and the risk of rosacea using MR and Bayesian colocalization analysis. Genetic evidence indicated that targeted modulation of IRF1 and SLC22A5 expression may serve as a potential strategy to reduce disease risk. Functional analyses further revealed that IRF1 drives disease progression by activating immune‐inflammatory pathways, while dysregulation of SLC22A5‐mediated metabolic processes may exacerbate the pathological phenotype. These findings not only identify novel molecular targets for the precise treatment of rosacea but also establish a theoretical framework for the development of targeted therapies, such as IRF1 inhibitors or SLC22A5 activators, thereby facilitating critical advancements in translating genetic research into clinical applications.

## Author Contributions

The authors would like to thank the following experts and individuals for their contributions: Xinrui Deng and Zhengchun Xie for their contributions to the data collection, analysis and writing of this paper; Huiyu Long and Jinyuan Xiao for his pivotal role in the primary data analysis; Professor Sui He for providing the idea and conclusions of the discussion for this paper. We thank all of them!

## Ethics Statement

This is a reanalysis of previously collected and public data; no additional ethics approval was needed.

## Conflicts of Interest

The authors declare no conflicts of interest.

## Supporting information


**Figure S1.** Manhattan plot for MR results (A) in blood (B) in sun‐exposed, and (C) in nonsun‐exposed. The *p* values are obtained using the IVW and Wald ration method, with the red dashed line denoting a significant MR estimate. MR, Mendelian randomization; sun‐exposed, sun‐exposed skin of the lower leg; nonsun‐exposed, sun not exposed suprapubic skin.
**Figure S2.** The SMR locus plots and effects plots for correlations of circulating IRF1 and SLC22A5 with rosacea. (A) IRF1, (B) SLC22A5.
**Figure S3.** Results of PheWAS analysis for IRF1 and SLC22A5. (A) Binary traits PheWAS association with IRF1, (B) binary traits PheWAS association with SLC22A5. The bottom dashed line represents the Suggestive line, and the top dashed line is the Significant line. Traits that exceeded the significant line were considered to be significantly associated with a gene.

**Table S1.** Data sources in our study sun‐exposed: sun‐exposed skin of the lower leg; nonsun‐exposed, sun‐not‐exposed suprapubic skin.
**Table S2**. The final list of druggable gene symbols used in this study.
**Table S3**. Genetic variants of eQTLs that were used in the discovery phase.
**Table S4**. Mendelian randomization analysis of eQTL in blood on rosacea.
**Table S5**. Mendelian randomization analysis of eQTL in skin tissue on rosacea. MR, Mendelian randomization; N, number of SNPs included in the MR analysis. CI 95, 95% confidence interval.
**Table S6**. Results of heterogeneity test.
**Table S7**. Results of MR Egger intercept horizontal pleiotropy test.
**Table S8**. Results of the Steiger directionality test. Both causal directions were tested. SNP r2, explained variance by the included SNPs on the exposure or outcome.
**Table S9**. Results of SMR and HEIDI test.
**Table S10**. Differentially expressed results of GEO analysis.
**Table S11**. The KEGG pathway analysis of IRF1‐centered network.
**Table S12**. The KEGG pathway analysis of SLC22A5‐centered network.

## Data Availability

The eQTL and GWAS data sets generated and/or analyzed during the current study are publicly available.
