# Supplementary material for: Integrated Genomic and GEO Data Analysis Reveals Therapeutic Targets for Rosacea
Source: J Cosmet Dermatol. 2025 Jul 10;24(7):e70334. doi: 10.1111/jocd.70334 (PMC12242367; doi:10.1111/jocd.70334)
Supplement: Supplementary file 1 — Figure S1. Manhattan plot for MR results (A) in blood (B) in sun‐exposed, and (C) in nonsun‐exposed. The p values are obtained using the IVW and Wald ration method, with the red dashed line denoting a significant MR estimate. MR, Mendelian randomization; sun‐exposed, sun‐exposed skin of the lower leg; nonsun‐exposed, sun not exposed suprapubic skin. Figure S2. The SMR locus plots and effects plots for correlations of circulating IRF1 and SLC22A5 with rosacea. (A) IRF1, (B) SLC22A5. Figure S3. Results of PheWAS analysis for IRF1 and SLC22A5. (A) Binary traits PheWAS association with IRF1, (B) binary traits PheWAS association with SLC22A5. The bottom dashed line represents the Suggestive line, and the top dashed line is the Significant line. Traits that exceeded the significant line were considered to be significantly associated with a gene. [file JOCD-24-e70334-s001.doc]

**
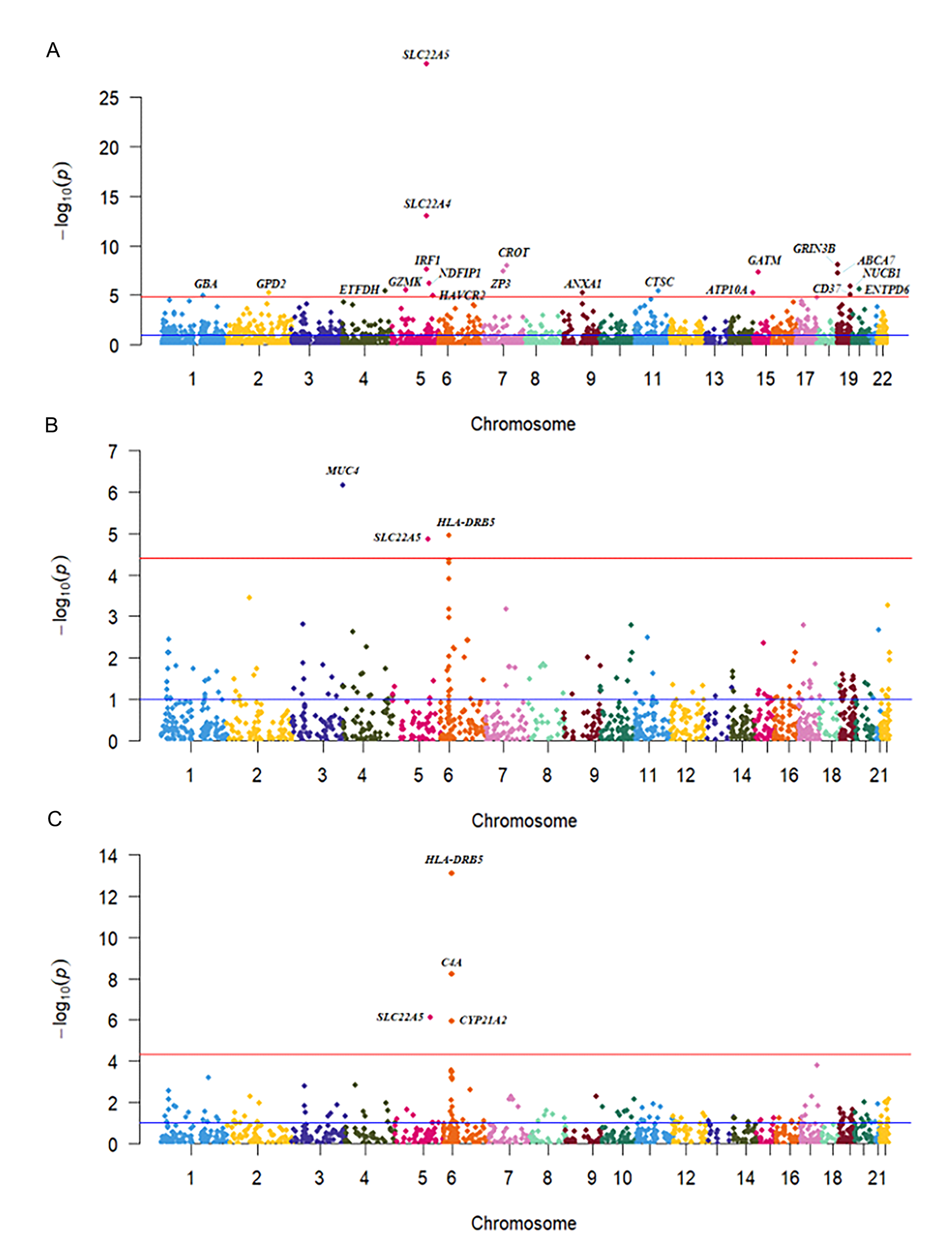
**

**Figure S1** Manhattan plot for MR results. A) in blood B) in sun-exposed C) in nonsun-exposed. The P-values are obtained using the IVW and Wald ration method, with the red dashed line denoting a significant MR estimate. MR: Mendelian Randomization; sun-exposed: sun-exposed skin of the lower leg; nonsun-exposed: sun-not-exposed suprapubic skin.

**A**


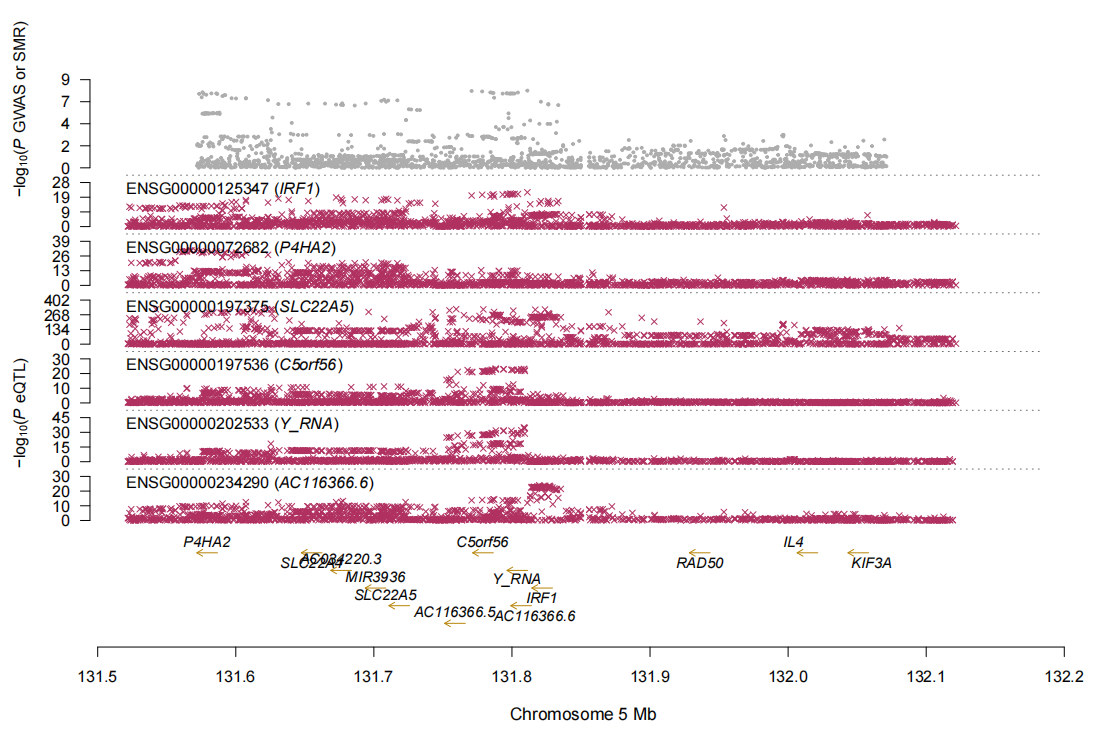


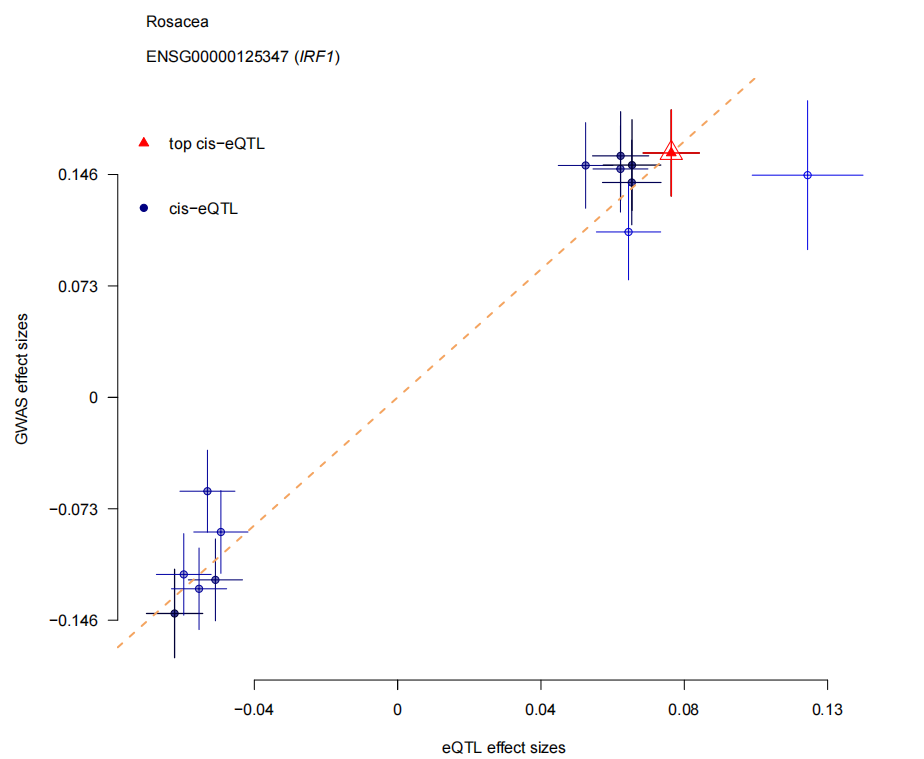


**B**
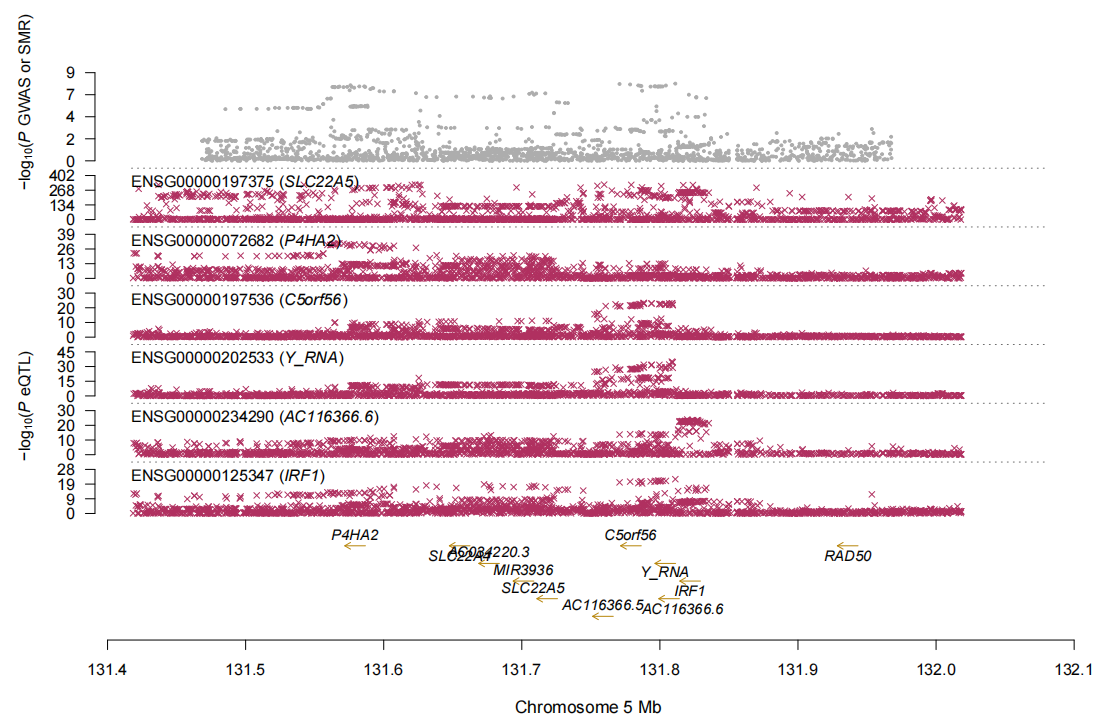


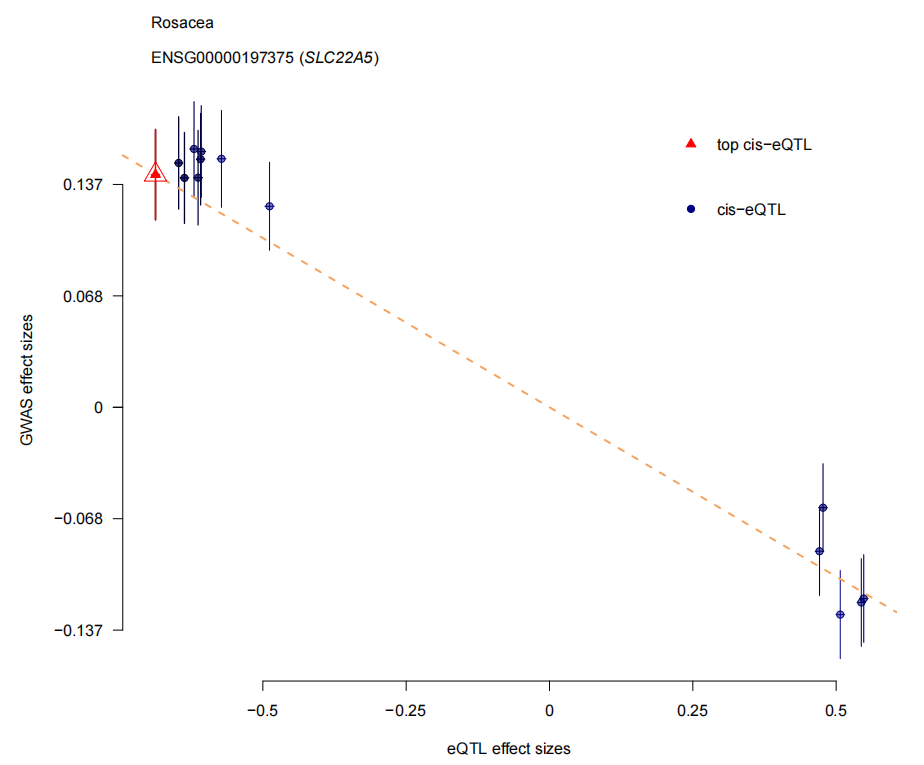


**Figure S2** The SMR locus plots and effects plots for correlations of circulating IRF1 and SLC22A5 with rosacea. A) IRF1 B) SLC22A5

**A**


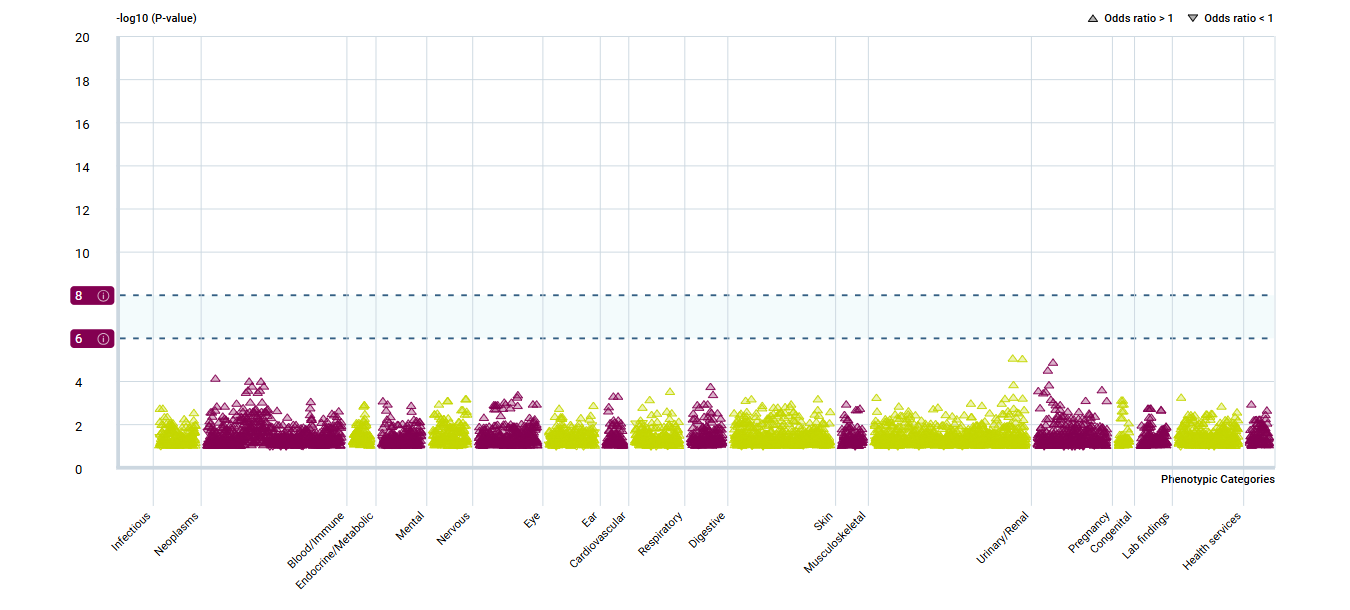


**B**


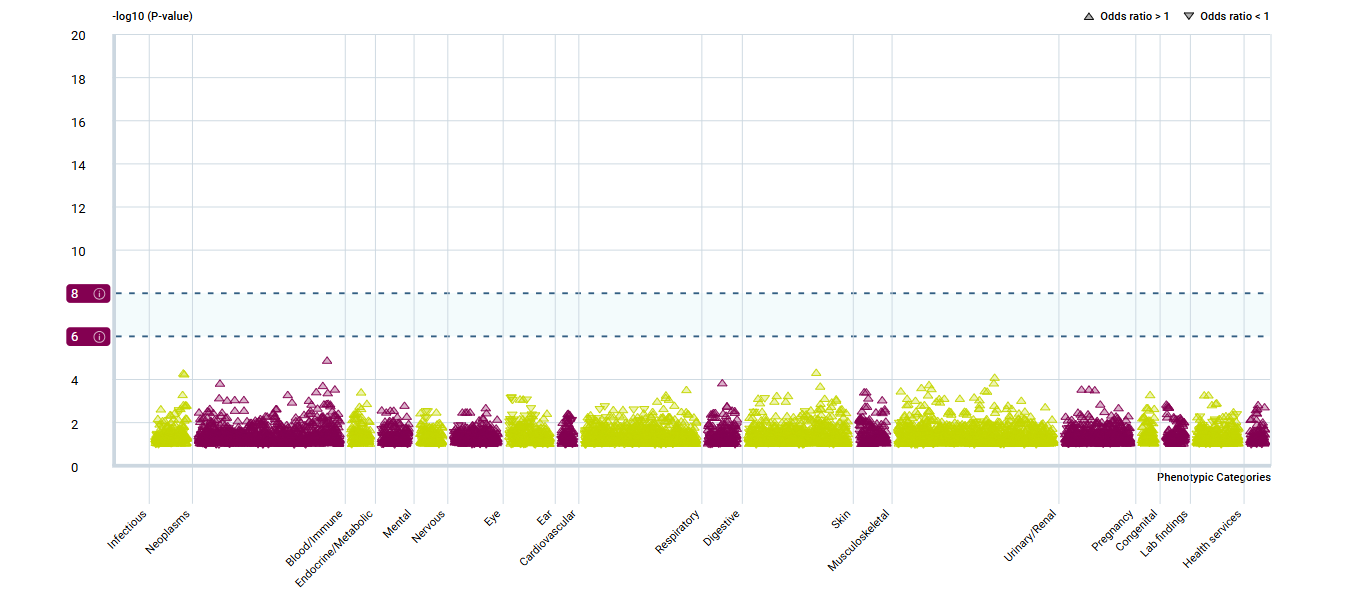


**Figure S3** Results of PheWAS analysis for IRF1 and SLC22A5. A) Binary traits PheWAS association with IRF1 B) Binary traits PheWAS association with SLC22A5 The bottom dashed line represents the Suggestive line and the top dashed line is the Significant line. Traits that exceeded the significant line were considered to be significantly associated with a gene.
